# What is the question?

**DOI:** 10.1186/s13059-019-1902-1

**Published:** 2019-12-19

**Authors:** Itai Yanai, Martin Lercher

**Affiliations:** 1 Institute for Computational Medicine, NYU Langone Health, New York, NY 10016 USA; 2 Institute for Computer Science & Department of Biology, Heinrich Heine University, 40225 Düsseldorf, Germany

This was [Stephen] Hawking’s central point in 1976 when he created something that came to be known as the information paradox. It was an extremely deep and important observation. It wasn’t important that Hawking didn’t get the right answer; he asked the right question. And this became a central debate that took twenty five years to resolve

Leonard Susskind (https://www.youtube.com/watch?v=2DIl3Hfh9tY, Minute: 13:40)

The single greatest misunderstanding about science by the public is that scientists solve problems; in reality, scientists are primarily concerned with creating them. We previously introduced François Jacob’s notions of *day science*, when we work with fixed targets in the lab or at the computer to solve problems, and *night science*, when our minds wander more freely to generate new ideas and find hidden connections [1]. It is certainly easier to imagine science as a logical, step-wise process. But it is the generation of a new question in the unpredictable and wandering process of night science that paves our way towards a discovery, effectively changing our perception of reality.

## What is your problem, Einstein?

Imagine being a fly on the wall of the office of Prof. Dr. Heinrich Friedrich Weber in the Polytechnic Institute in Zürich in 1900.

“What can I do for you, Mr. Einstein?” asks the professor sternly as he looks at one of his least favorite students.

“Professor” the bold student begins, “what are the greatest open questions of theoretical physics? I wish to tackle them.”

“Well, young man, as you would surely know had you regularly attended my lectures, there are three major unsolved problems today. I don’t think your talents are up to the task, but I will humor you with a retelling: How do we have to change our concept of time so that Maxwell’s equations are no longer in contradiction with the observed constancy of the speed of light? How can the absorption and emission of light in discrete packages avoid inconsistencies in our concept of black body radiation? And finally: How can gravity be understood as deformations in space and time?”

Equipped with these questions, young Einstein rushes back to his lonesome desk. The curious scientist tackles them one by one, braving each logical step as it comes, each leading him undeterred to elegant conclusions. He solves all three problems by the age of 40, transforming himself into the iconic scientist we know today. This is the highroad of scientific progress: the leaders of a field identify the major open questions—the knowledge gaps in the “brick wall” of science—and then creative individuals around the world brood over them until someone derives the answer.

Aiming to accelerate this scientific process, it is not uncommon to find public lists of open scientific questions. Panels of cancer biologists list provocative questions singled out for funding [2]. Mathematicians have a list of seven unsolved “Millennium” problems, with a million dollar prize for each solution [3]. The contributors to Wikipedia provide lists of open questions for 14 different disciplines, including physics, chemistry, biology, medicine, and neuroscience. So can we reasonably expect the leading scientists of each discipline to gather 10 years from now, nominate the bright minds that answered those questions for prizes and medals, and compile the next top ten lists? Surprisingly—or not, as we will argue—if you compare a list of the great discoveries in the life sciences over the 25 years leading up to 2015 with the list of questions provided early on in this period, you notice very little overlap (Table 1).

**Table 1:** A comparison of the top 5 open problems in the life sciences posed in 1997 and the 5 biggest discoveries of the past 25 years listed in 2015

1997: the top most outstanding problems in the life sciences [4]	2015: the 5 biggest discoveries of the past 25 years [5]
1. What was the origin of life?	1. RNA interference discovered (1998)
2. What is the genetic and molecular basis of neural specificity?	2. Dolly the sheep becomes the first adult mammal cloned (1996)
3. How are genes regulated in animals and plants?	3. Human genome mapped (2000)
4. Topics in developmental and behavioral biology	4. Stem cells created from mature skin cells (2007)
5. How can we predict protein folding and the three-dimensional structure of proteins from amino acid sequences?	5. Robotic limbs fully controlled by the brain (2009)

And as you might have guessed, Einstein did not have a top three list of open questions to start with. What he did have were topics in the form of puzzling observations, puzzling primarily to himself. Let us take the first one as an example. When Einstein was still in school, he arrived at a fascinating paradox: if you imagined traveling parallel to a light beam at the speed of light, it should look like a standing, oscillating wave—but that would contradict Maxwell’s equations, which otherwise seemed so perfect at explaining the properties of electromagnetic radiation. For years, Einstein tried to find a way to modify Maxwell’s equations so that things would fall into place. He failed, again and again, until one night, coming home from a visit to a friend to whom he had complained about his failure, it dawned on him: it was not Maxwell’s fault. It was time’s. What if our notion of time itself was incorrect? In a moment when he was not consciously wrestling with equations but left his mind wander freely—in other words in a bout of night science—Einstein had finally arrived at the very question that was the key to his conundrum: was there a way to change our concept of time that would make things fit? Einstein was not given the question. He discovered it.

The trouble with trying to solve questions posed by communities is that all the good ones are gone, especially if they *can* be answered. Why then do not we see the scientists around us spend their nights hunting for questions? Instead, it seems that having a clear question is a scientist’s natural state of mind; after all, the storyline of almost every scientific paper starts with a clearly defined question and then proceeds directly to the answer. In reality, the way scientists retell their discoveries may reflect much more how humans communicate knowledge than how those discoveries were actually made. It is not just that humans have always loved a good story [6]; a linearly structured exposition with logical steps is indeed the most effective way of instruction. Hidden behind the storylines of our papers, we may have spent long nights wandering around for questions. But once we stumbled upon the right one, it was transformative, often almost completely erasing our prior goals.

## Unknown unknowns

We often see knowledge as a wall of information: individual pieces of knowledge fit together like bricks within the wall, summarizing what is known on a particular topic. This metaphor suggests that the way to advance science is to extend this wall of knowledge, strengthening it and thereby increasing its explanatory power, or extending it beyond the edges of a text book. A hole in the wall is seen as a “knowledge gap,” and we can “flesh out” existing theories by closing such gaps. And indeed, addressing a specific problem may often lead to knowledge that fits squarely within the confines of a wall of knowledge.

But this picture gives a false sense of the structure and rigidity of knowledge and its accumulation. The nature of discoveries is that they are unexpected: they may not fit neatly into our existing edifice of knowledge. Although the research may be originally motivated by a perceived gap, the knowledge resulting from the discovery may in fact not complete any part of the wall but instead may lead to the construction of a completely new and unexpected area: we may be forced to build a new wall orthogonal to the first, or even to tear down parts of the existing structure. This is an uncomfortable concept for many of us, who would prefer a tidy and beautiful universe, where a rational process helps us to illuminate the world. And yet, the most interesting unknowns of science are unknown unknowns—gaps that we were not even aware of before chancing upon them.

A truly new question, as an unknown unknown, is not predictable, and generating it requires night science in addition to our day science work. This aspect of the research process is often hidden by the work that follows the invention of the question. In some cases, scientists may spend many years on answering that question, as in the quote on Stephen Hawking at the outset. And while scientists are systematically taught the process of day science—experimental design and controls—they are typically immersed only slowly in the depths of night science: A student joining a lab is often presented with a hypothesis to work on and may see science as a hypothesis-testing endeavor. Many young postdocs have been told that as a PhD student their job was to answer questions—now they have to discover their own unknown unknowns.

Community-generated questions such as those in the left columns of Tables 1 and 2 are typically so general that they do not provide a new direction towards an answer. Answering one of them almost always requires a rephrasing, a refocusing of the original question, which exposes a new aspect of the problem and only becomes possible after an insight into the phenomenon at hand. As an example, “Does the microbiome affect a tumor’s growth?” is a valid question, but it can only serve as a starting point for our explorations. After some initial analyses and much subsequent night science, we might go back and ask, for example, “Does a tumor manipulate the microbiome as a kind of co-conspirator?”, or “Can bacteria become intra-cellular components of a cancer cell?”. These may lead to hypotheses that are testable and novel.

**Table 2:** Rephrased questions that led to scientific breakthroughs

Lead scientist	Original question	Refocused question
Marie Curie	Where does the “radioactivity” (a term later coined by Curie herself) come from?	How does the radioactive activity depend on the form and quantity of the uranium in a given sample? [7]
Charles Darwin	Why do similar (though not identical) species occupy geographically related niches?	Do species split to form distinct species when they are geographically segregated and adapting to different environments? [8]
Kurt Gödel	Can you make a complete, contradiction-free formal system of all mathematical theorems?	Can you use number theory to construct statements that are neither provable nor disprovable in such formal systems? [9]
Barbara McClintock	How are phenotypes controlled on the molecular level?	How do genes switch on and off certain characteristics of an organism? [10]
Francisco Mojica	Why do bacteria have CRISPR elements?	Can we learn about the function of CRISPR by looking at sequence similarities of the spacers to known sequences? [11]

Sometimes, such new questions will not even be posed in response to a specific public question, yet may lead to answering it in an unexpected way. Our ignorance about a certain topic often provides a fertile ground for novel questions [12]. Discovering the question follows from immersion in a particular topic. Francisco Mojica, for example, provided a radically new hypothesis for why bacterial genomes have a structure that previous researchers had termed “clustered regularly interspaced palindromic repeats” (or CRISPR), separated by evenly sized “spacers” of apparently random DNA. Before Mojica’s work, not many scientists were interested in these peculiar structures, and the problem of CRISPR elements could have been stated as “Why do bacteria have CRISPR elements?”. However, this question is too general to be solved, lacking any hints at where to look for the answer. The inconspicuous spacers were largely ignored. Mojica, however, asked [11]: what does the similarity of the spacers to known DNA sequences tell us about their function? Again, it was the question that led the way, generated in night science but requiring rigorous day science for its answer: the spacers are copies of viral sequences, guiding an adaptive bacterial immune system towards their destruction. Table 2 lists more examples of questions whose refocusing led to breakthroughs.

## Telling stories

As the Susskind quote about Stephen Hawking above shows, if a scientist proposes an important question and provides an answer to it that is later deemed wrong, the scientist will still be credited with posing the question. This is because the framing of a fundamentally new question lies, by definition, beyond what we can expect within our frame of knowledge: while answering a question relies upon logic, coming up with a new question often rests on an illogical leap into the unknown—the hallmark of night science.

**Fig. 1:**
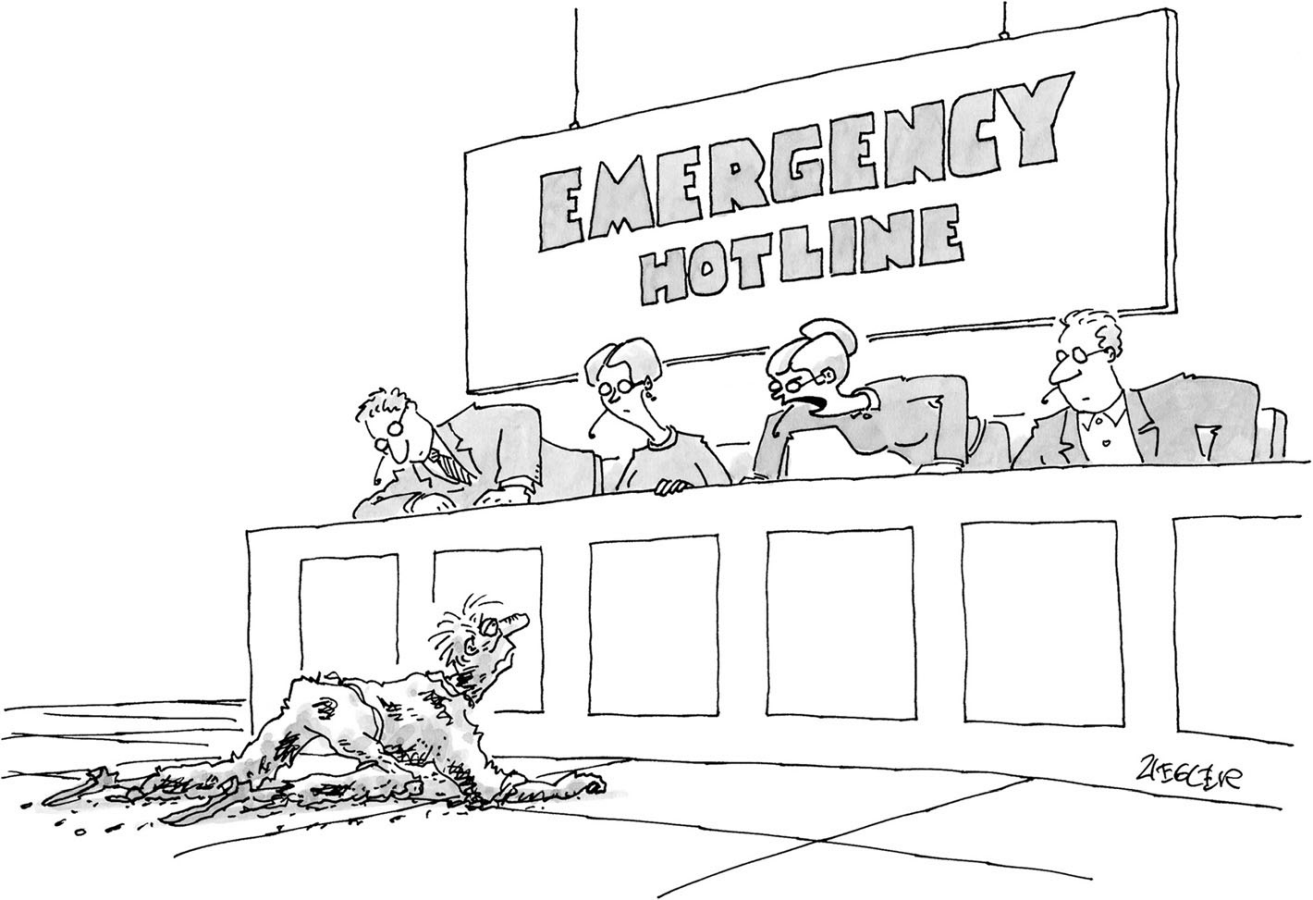
A New Yorker cartoon contest. Can you think of some funny caption to make sense of the cartoon? (Credit: www.JackZiegler.com, licensed from the New Yorker issue May 9, 2005)

**Fig. 2:**
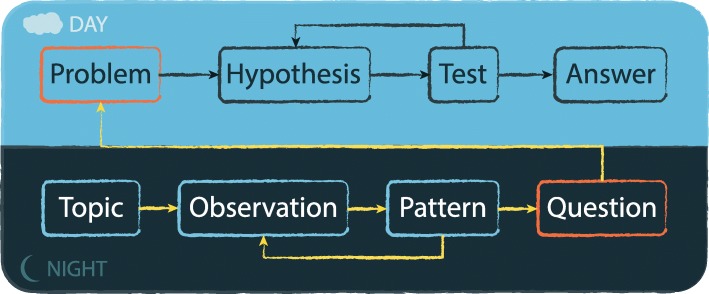
The perceived (day science) and hidden (night science) view of the scientific method. (The caption for the winning cartoon in Fig. 1 is “Neither the time nor the place, Doug!”)

Why, then, does it not seem this way? Why do questions appear secondary to answers? It may be because a new question is so powerful that it transforms our reality. A new question tends to erase its own origin; it is hard to imagine that there ever was a time when the question was not there. The effort immediately shifts to figuring out the answer to the new aspect of reality illuminated by the question. To get a sense of this, consider the weekly New Yorker Cartoon Contest, where you can propose a funny caption for a caption-less cartoon. This is a difficult challenge, as appreciated by anyone who has attempted this (try it yourself in Fig. 1). The minute you read someone else’s caption though, you are tied to this particular solution (there is one hidden in the caption of Fig. 2). Likewise, a new scientific question seems obvious once stated (such as “What can you learn from the similarities of CRISPR spacers to known DNA sequences?”), but that should not lead us to think that the question’s introduction was obvious, too.

Finding the question can be fun, as in thinking of a cartoon caption. But it can also be extremely difficult psychologically. Scientists are often expected by the public to know it all, and yet, “feeling stupid” is a common mode of operation for us [13]. Science is the art of dealing with things we do not know enough about. As Wernher von Braun, the father of German and US rocket programs, phrased it: “Research is what I’m doing when I don’t know what I’m doing.” Science is humbling in this way. For young scientists, it is often very difficult to understand that it is perfectly normal to not know the answer—or even the question. Learning to embrace this uncertainty is part of our maturation as scientists.

Uri Alon has an intuitive image to describe the process of re-finding our questions [14]. Given what we know about a given topic “A,” a researcher predicts that it should be possible to arrive at point “B,” a scientific destination that seems interesting—a hypothesis. However, the plot inevitably thickens over the course of the research project, and new hurdles force the scientist into a meandering path. Soon, the researcher is lost, having lost sight of the start point (which suddenly seems shaky) and end point (which appears unreachable). Uri calls this “being in the cloud”—you have lost your original question, but the reason why this has occurred is strange and thus potentially exciting and itself worthy of study. From inside the cloud, the situation may seem desperate, but Uri sees the cloud as the hallmark of science: if you are in the cloud, then you might have stumbled upon something non-obvious and interesting. “I’m very confused” a student would tell Uri, to which he would reply, “Oh good - So you’re in the cloud!” Eventually, a new question that arose inside the cloud may lead the way to an unexpected destination “C.”

## Embracing uncertainty

The scientific method is often perceived as a simple sequence that leads from a problem to an answer, possibly through long iterations of modified hypotheses. But our reality is much less structured: it often starts with a topic and some observations, leading to the finding of patterns and questions about those patterns, possibly long before we have any explicit hypothesis or any direct tests (Fig. 2). And even if a project starts out with a very specific hypothesis, in our experiences, it still generally arrives at a very different point than expected.

In some way, then, night science may be most productive when it has no agenda, when there are no particular questions it is trying to reshape or resolve. When the scientist does not have a hypothesis, she is free to explore, to make connections. In some sense, any kind of expectation on how things are to behave—a hypothesis—is a liability that could obstruct a new idea that awaits our discovery. Once night science elucidates and reframes this question, the researcher can use the full power of day science to solve it. In this sense, a major discovery is typically both the solution and the problem.

Much of basic, curiosity-driven science is exploration, and night science is a fundamental part of that; yet funding bodies often demand that research must be hypothesis-driven. But while some part of night science can be done with the help of an armchair and some good coffee, other parts require the exploration of large and complicated data sets. If no funding is provided for such endeavors, the generation of new questions may be stifled, hindering scientific progress: in science, the problem that is eventually solved is often not the one that was initially sought out.

To be sure, every one of us spends a lot of their time solving questions that have already been posed. For example, we might work out the particular regulatory structure of a gene or the evolution of a gene family. Often, the hope is that this immediate problem, once solved, will lead to a new and exciting question. A case in point was the sequencing of the human genome: the initial scientific question was clear (“What is the DNA sequence of a human genome?”), but the really exciting questions about our genome biology arose only afterwards.

If an idea is truly unexpected, then we could not have arrived at it solely through existing questions; instead, we had to navigate through night science, moving from disparate observations to previously unknown questions. It is freeing and exhilarating to embrace this uncertainty, to fly right into the heart of the cloud, even if we may feel stupid and lost there. Night science, that realm where questions and ideas are born, appears so mysterious that it is often not described at all. But it is our premise that there are patterns to it, and this is what will occupy us in the following installments of this mini-series.
